# Portion Estimation, Satiety Perception and Energy Intake Following Different Breakfast Portion Sizes in Healthy Adults

**DOI:** 10.1111/nbu.12733

**Published:** 2025-02-05

**Authors:** Kinga Kwiecien, Lourdes Santos‐Merx, Tarsem Sahota, Helen Coulthard, Mariasole Da Boit

**Affiliations:** ^1^ Leicester School of Allied Health Sciences De Montfort University Leicester UK; ^2^ School of Health, Sport and Food University College Birmingham Birmingham UK

**Keywords:** appetite regulation, breakfast, energy intake, portion size, satiety, snacking

## Abstract

Expected satiety is a key element in predicting meal portion size and food consumption; however, how this can be affected by different breakfast portion sizes is unknown. The study examined the impact of different breakfast portions on satiety, portion size, and energy intake and comprised an online survey and an experimental intervention. Sixteen adults (9 women, BMI: 24.9 ± 4.3 kg/m^2^) rated images of three portion sizes (small, standard, large) of the same breakfast using an ordinal scale. Subsequently, they were asked to self‐prepare and consume ad libitum the three breakfast portions in a randomised order on different days and to complete a food diary. Satiety and portion size perception were re‐measured upon consumption of each breakfast. For both the visual image and breakfast consumption, the small breakfast portion was rated as the smallest and least filling, while the large portion was rated as the largest and most filling (*p <* 0.05). When consuming the small breakfast, participants reported being hungrier and less full between breakfast and lunch (*p <* 0.05) and had a higher energy intake from lunch onward, due to more snacking (*p <* 0.05). However, the total daily energy intake was not different among the three breakfast portion sizes. Individuals seemed accustomed to predicting satiety and portion size from images. The consumption of the small breakfast was judged as not filling enough and was accompanied by a higher energy intake via energy‐dense snacks. Based on these preliminary findings, breakfast size reduction may lead to unhealthy compensatory energy intake by snacking on energy‐dense foods.

## 1 Introduction

Obesity is a widespread condition (Blüher 2019; WHO 2022) and a major risk factor for many diseases, such as cardiovascular diseases, and more recently for COVID‐19 morbidity and mortality (Albuquerque et al. 2017; Blüher 2019; Popkin et al. 2020). The two main factors contributing to weight gain are chronic increased daily energy intake and reduced physical activity (Panuganti, Nguyen, and Kshirsagar 2022).

Breakfast has been considered the most important meal of the day (Chowdhury et al. 2016; Gibney et al. 2018; Rong et al. 2019). Within the UK dietary recommendations, breakfast should consist of around 20% of total daily energy and nutrient intake (Public Health England 2017). Moreover, ‘Do not skip breakfast’ is one of eight tips for healthy eating (Public Health England 2018); however, guidance on what constitutes a standard breakfast in terms of food items and portion size is scarce (Gaal et al. 2018). Nonetheless, irrespective of which meal, over the years there has been an expansion in both self‐selected and food manufacturers'/outlets' portion sizes (Wrieden, Gregor, and Barton 2008). There are several explanations behind this, including difficulties in estimating food portion sizes and when considering a unit of food (e.g., a sandwich), the amount to be consumed per eating occasion regardless of the recommended amount (Blake, Guthrie, and Smiciklas‐Wright 1989; Steenhuis and Poelman 2017). A number of studies demonstrate a positive correlation between an increase in portion sizes and total daily energy intake, also known as ‘the portion size effect’ (Rolls, Morris, and Roe 2002), and other studies suggest a potential link between portion size and obesity (Livingstone and Pourshahidi 2014). Nevertheless, if an increase in meal portion size leads to increased energy intake, it is not clear whether a reduction in portion size below what is considered a ‘standard’ food portion may or may not promote compensatory energy intake. A laboratory‐based study by Haynes, Hardman, Halford, Jebb, Mead, et al. (2020) found that a reduction in both lunch and dinner portion size, below the participants' perceived ‘normal’ size, did not contribute to overcompensation of food throughout the day and led to a decrease in total daily energy intake (Haynes, Hardman, Halford, Jebb, Mead, et al. 2020). Similarly, a reduction in breakfast size by both 20% and 40% showed no differences in compensatory energy intake for the remainder of the day (Lewis et al. 2015), although this has not always been observed. French et al. (2014) reported no significant decrease in total daily energy intake in the reduced lunch portion group compared to the ‘typical’ lunch portion group over 6 months. The difference in these findings might be due to different experimental conditions (laboratory vs. free‐living), the duration of the intervention, and/or the size of reduction in portion size. Nonetheless, despite these conflicting findings, it seems that manipulation of portion sizes might be a potential strategy to promote weight management. However, more intervention studies are needed to better understand the impact of portion sizes on daily energy intake.

Appetite regulation is a complex interplay between psychological and physiological determinants, which regulate satiety and satiation (Casanova et al. 2019). Recent studies have highlighted the role of cognitive activity in pre‐determining the fullness at the end of a meal (Brunstrom 2014; Robinson, te Raa, and Hardman 2015). For instance, Wilkinson et al. (2012) asked volunteers to estimate satiety and ideal portion size of three screen‐based images of different foods, one of which was later consumed. The authors reported that ‘expected satiety’ greatly predicted ideal and self‐selected meal size and food consumption (Wilkinson et al. 2012). From these studies, it is evident that pre‐meal planning is an important factor in determining portion size selection and food consumption, although currently there is a lack of research on how different breakfast portion sizes affect ‘expected satiety’. More clarity on the role of breakfast portion size on ‘expected satiety’ and daily energy intake is paramount to better understand the possible implications on energy balance.

This study aimed to investigate the effects of three different portions of the same breakfast (standard, reduced by 50%, and increased by 50% of the standard breakfast energy reference) on portion size estimation, satiety and energy intake. The first aim was to compare ‘expected satiety’ and portion size estimation of visual images of the different breakfast portions with portion size evaluation and satiety after the actual consumption of the three breakfast portions. The second aim was to assess the effect of the three breakfast portions on daily energy intake using a randomised repeated measures design.

Our first hypothesis was that visual portion size estimation will differ from portion size rated after breakfast consumption, while ‘expected satiety’ of visual images will not differ from satiety perception after breakfast consumption. Our second hypothesis was that daily energy intake will not be compensated throughout the day after the reduced breakfast size, leading to a reduction in total daily energy intake.

## 2 Methods and Materials

### 2.1 Participants

Participants between 18 and 80 years of age were recruited through social media (Facebook and LinkedIn) and word of mouth. The inclusion criteria were being a regular breakfast eater (4 or more days a week), not taking insulin, not engaging in a weight loss programme, not being medically diagnosed with eating disorders, and not being allergic to or disliking the foods featured in the study. Screening data acquired from the EAT‐26 questionnaire, questions on breakfast habits and anthropometric measurements were used to assess eligibility. A G power a priori calculation with 3 groups (small, medium and large breakfast portions) and two measures (portion size estimation and expected satiety) indicated that the sample should be a minimum of 13 participants (power = 0.8, *α* = 0.05). All participants were provided with an online participant information sheet and provided informed consent prior to the beginning of the study, for both parts of the study. This research study was conducted according to the Declaration of Helsinki and approved by the Research Ethics Committee at De Montfort University in Leicester (24/02/2020, ref.: 3493).

### 2.2 Design

**FIGURE 1:**
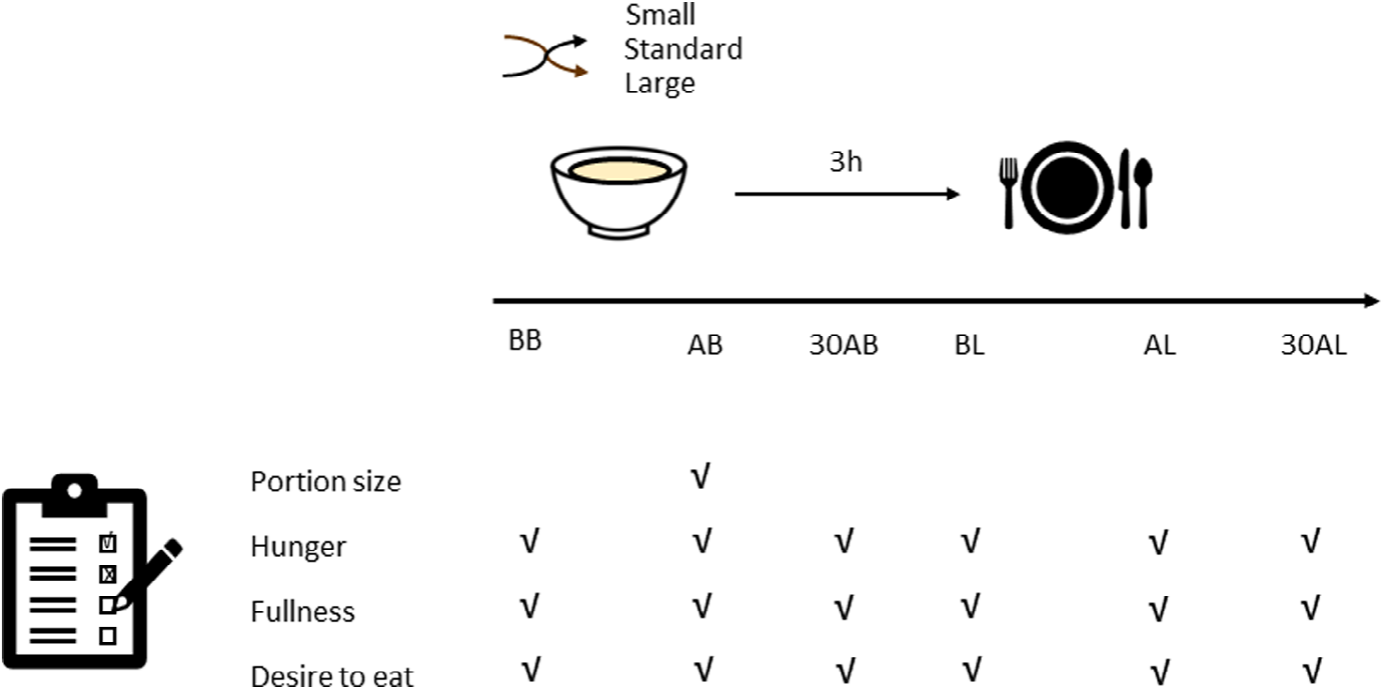
Schematic representation of the randomised breakfast consumption study (study part 2). BB (before breakfast), AB (after breakfast), 30AB (30 min after breakfast), BL (before lunch), AL (after lunch) and 30AL (30 min after lunch). Portion size question: ‘How large do you consider this portion size?’, hunger question: ‘How hungry do you feel now?’, fullness question: ‘How full do you feel now?’, desire to eat: ‘How strong is your desire to eat now?’; breakfasts: small, standard, and large.

This study was divided into two parts: an online survey (part 1—breakfast portion size visual evaluation), and an experimental intervention (part 2—experimental breakfast consumption). After completion of the first part of the study, participants were invited to undertake the second part of the study (Figure 1). Due to COVID‐19 restrictions, the second part was done in free‐living conditions, enabling the assessment of eating patterns in a real‐life setting (Gough et al. 2021).

A within‐subject experimental design was adopted for both data collection points in the study. To address the first hypothesis, the dependent variables were portion size estimation and ‘expected satiety’ rating. There were two factors: breakfast size (small, standard and large) and the mode in which the portion sizes were estimated (image vs. actual). To address the second hypothesis, the two factors were the time of rating with five levels (before breakfast, after breakfast, 30 min after breakfast, before lunch and after lunch), and the breakfast portion size with three levels (small, standard and large). The dependent variables were fullness, desire to eat and hunger. Additionally, total daily energy intake, total daily energy intake without breakfast, lunch kcals, dinner kcals and snack kcals were dependent variables in relation to one within‐participant factor, breakfast portion size (small, standard and large).

### 2.3 Experimental Materials and Procedure

#### 2.3.1 Breakfast Portion Size Visual Evaluation (Part 1)

**FIGURE 2:**
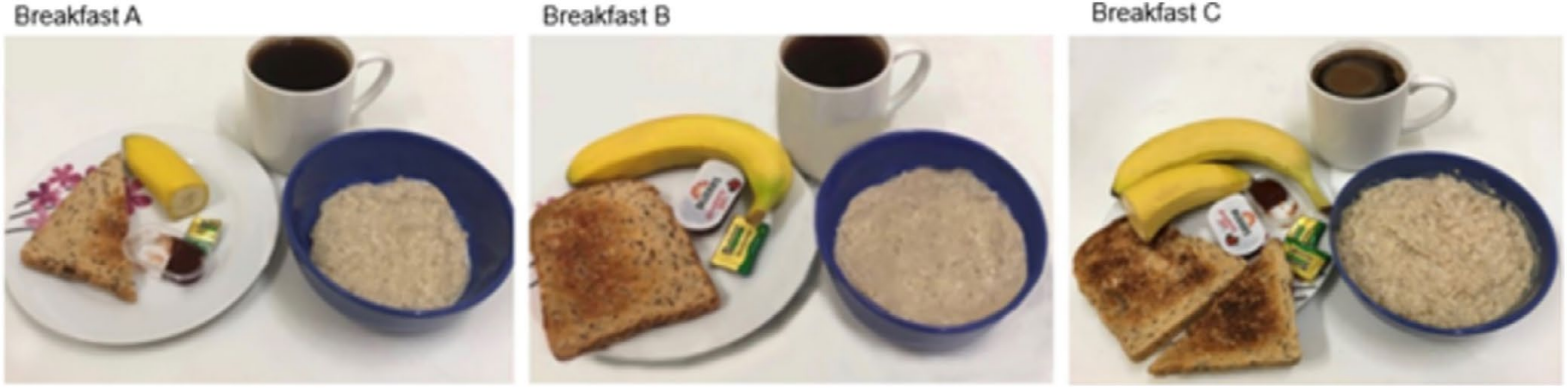
Food composition of the three different breakfast portion sizes. Small (A), standard (B) and large (C) breakfast.

In the online survey, a photo of three different sizes of the same breakfast was included (Figure 2). The sizes (with the percentage of the 2000 kcal daily Reference Intake and calories in brackets) were as follows: small breakfast (10%; 200 kcal), standard breakfast (20%; 400 kcal) and large breakfast (30%; 600 kcal). The size of the breakfasts was based on the Reference Intake of a 2000 kcal diet for an average female adult, used for labelling purposes, with a nutritional composition of around 50% carbohydrates, 35% fat, and 10% protein (British Nutrition Foundation 2021; Gaal et al. 2018; Public Health England 2016). The breakfast included porridge oats, semi‐skimmed milk, banana, wholemeal toast, butter or jam, and a cup of coffee, with the respective amounts shown in Table 1.^^ Participants were not informed of the energy value and nutritional content of each breakfast.

**TABLE 1:** Food and macronutrient composition of the small, standard and large breakfast.

Food items	Small (200 kcal)	Standard (400 kcal)	Large (600 kcal)
Porridge oats (g)	13.5	27	40.5
Semi‐skimmed milk (mL)	90	180	270
Apple/banana (g)	76/45	145/84	221/129
Wholemeal bread (g)	20	40	60
Butter/reduced sugar jam (g)^a^	3.1/10	6.2/20	9.3/30
Optional tea/coffee without sugar (mL)	250	250	250
Carbohydrates (g)^b^	35	69	104
Protein (g)^b^	8	16	23
Fat (g)^b^	5	9	14

^a^ Only if preferred to butter; suggested amounts of butter and jam provided the same energy value.

^b^ Macronutrient contents for the choices of apple or banana and butter or jam were averaged.

For each photo, volunteers were asked two questions. The first question measured portion size estimation: ‘*For yourself, how would you consider the portion size of breakfast marked in red to be?*’. This was designed to test how participants viewed the size of each breakfast when presented with a photographic representation of food. Participants had to indicate their answer on an ordinal scale that varied between ‘extremely small to extremely large to me’ valued zero and 10, respectively. The second question measured expected satiety: ‘*If you were to consume this breakfast, how full would you be?*’. This was created to establish the feeling of fullness they thought they would experience after consuming each portion of breakfast. For this question, the anchors of the ordinal scale were 0 (not at all) and 10 (extremely).

#### 2.3.2 Experimental Breakfast Consumption (Part 2)

Participants were given clear instructions on how to prepare the breakfasts, with servings, food items and cooking procedures included. They were instructed to eat as much as they wanted of the breakfast made, and if they had any leftovers, they were asked to take a photo and send it to the researchers along with the amount not eaten. The three breakfasts were consumed on alternate days, in a randomised order and after an overnight fast.

The breakfast consisted of the same food products and amount used for the photos shown in Part 1, but with the option of an apple instead of a banana, the choice of either butter or reduced sugar jam, and a cup of tea or coffee, with optional sweeteners and their usual amount of milk. This was to allow for taste preferences and available foods. The same choices were maintained for all three breakfasts. To ensure consistency with the online study, participants were not informed of the energy value and nutritional content of each breakfast.

On the breakfast intervention days, participants were asked to refrain from moderate and vigorous exercise and avoid food consumption, except water, between breakfast and lunch. They were also asked to eat lunch around 3 h after breakfast, and from lunch onward they were allowed to consume food without restrictions given. Right after consuming each breakfast, participants were asked to follow a provided link and access an online form to answer two questions: one on portion size and one on satiety, as in Part 1. The first question measured portion size estimation: ‘*How large do you consider this portion size?*’; while the second question measured actual satiety, ‘*Now you have consumed this breakfast, how full do you feel?*’. Furthermore, volunteers were asked to complete on the same online form additional repeated ordinal scales before breakfast (BB) and after breakfast (AB), 30 min after breakfast (30AB), and before lunch (BL), immediately after lunch (AL), and 30 min after lunch (30AL) to measure hunger, fullness, and desire to eat. The questions were as follows: ‘*How hungry do you feel now?*’ ‘*How full do you feel now*?’, ‘*How strong is your desire to eat now?*’. For portion size, the anchors were ‘extremely small for me’ and ‘extremely large for me’, while for the remaining questions, the anchors were ‘not at all’ and ‘extremely’, valued zero and 10, respectively. These scales were used to track changes throughout the day, as utilised by others (Lewis et al. 2015).

#### 2.3.3 BMI

Participants' height and weight were self‐reported by participants. Height and weight were converted into BMI using the standard formula (kg/m^2^).

#### 2.3.4 Food Composition and Energy

During each breakfast intervention day, volunteers recorded their food intake using an online food diary. Participants were asked to report the exact intake of their food as accurately as possible, following a visual portion size guide (British Nutrition Foundation 2019), including weighing ingredients and product label information. Total daily energy intake, total daily energy intake without breakfast, lunch kcal, dinner kcal, snack kcal, and macronutrient composition were calculated using MyFitnessPal (Evenepoel et al. 2020).

### 2.4 Statistical Analysis

All statistical analyses were performed using IBM SPSS Statistics software (v26, IBM Business Analytics; IBM, Hampshire, UK). Shapiro–Wilk's test was used to check data normality. Two 2 × 3 factorial repeated measure ANOVAs, with Bonferroni post hoc tests, were carried out to examine differences in (1) portion size estimation and (2) expected satiety, according to the factors of delivery mode (visual image or actual breakfast) and breakfast portion size (small, standard, large). A further three 3 × 5 repeated measure ANOVA, with Bonferroni post hoc analyses, were used to analyse hunger, fullness, and desire to eat ordinal scores at five different time points throughout the day (BB [before breakfast], AB [after breakfast], 30AB [30 min after breakfast], BL [before lunch] and AL [after lunch]). One‐way ANOVA was used to analyse the area under the curve (AUC) for hunger, fullness and desire to eat ordinal scores of the 3 breakfast conditions. AUC was calculated using the trapezoid method. Differences in total daily energy intake, total daily energy intake without breakfast, lunch kcal, dinner kcal and snack kcal according to the size of breakfast (small, medium and large) were analysed using five separate one‐way repeated measure ANOVAs. Data are expressed as mean ± SD. Statistical significance was accepted as *p* < 0.05.

## 3 Results

A total of 18 adults residing in the UK were enrolled in this study. Two participants dropped out for undisclosed reasons and, therefore, a total number of 16 adults (7 men and 9 women, age: 39.9 ± 13.6 years, stature: 169.8 ± 9.6 cm, body mass: 71.7 ± 13.5 kg, BMI: 24.9 ± 4.3 kg/m^2^) were included for the analysis.

### 3.1 Breakfast Portion Size and Satiety Rating According to Portion Size and Mode of Evaluation

Analysis of the differences in portion size estimation and expected satiety according to the portion size (small, standard, and large) and mode of evaluation (visual image vs. post‐breakfast consumption) was carried out (Table 2). There was a significant main effect for breakfast portion size (*p* < 0.001) on portion size estimation. Bonferroni post hoc tests revealed that all breakfasts received a significantly different score (*p <* 0.001) from the others, with the small breakfast considered to be the smallest and the large breakfast the largest. The same pattern of differences was found for expected satiety; there was a main effect of breakfast portion size (*p <* 0.001) on satiety ratings, with the small breakfast considered to be the least filling and the large breakfast the most filling (*p <* 0.05). There was no main effect (*p >* 0.05) for the mode of evaluation (visual image vs. post‐breakfast consumption), nor was there an interaction between portion size and mode of evaluation (*p >* 0.05) for both portion size estimation and expected satiety.

**TABLE 2:** Ordinal scale ratings of portion size and satiety in relation to the visual evaluation and upon consumption of the small, standard, and large breakfasts in healthy individuals (*n* = 16).

	Portion size estimation			Satiety		
	Small	Standard	Large	Small	Standard	Large
Visual evaluation	4.4 ± 1.7^†^	6.3 ± 1.4^‡^	8.8 ± 1.3^§^	5.9 ± 2.2^†^	7.7 ± 1.6^‡^	9.2 ± 1.2^§^
Consumption	3.3 ± 1.8^†^	5.9 ± 1.7^‡^	8.6 ± 1.5^§^	6.2 ± 2.0^†^	8.1 ± 1.3^‡^	9.1 ± 0.9^§^

*Note:* Data are presented as group mean ± SD. Visual evaluation of portion size estimation question: ‘For yourself, how would you consider the portion size of breakfast marked in red to be?’, anchors: 0 (extremely small) and 10 (extremely large). Portion size estimation upon consumption question: ‘How large do you consider this portion size?’, anchors: 0 (extremely small) and 10 (extremely large). Visual evaluation of expected satiety question: ‘If you were to consume this breakfast, how full would you be?’, anchors: 0 (not at all) and 10 (extremely). Satiety upon consumption question: ‘Now you have consumed this breakfast, how full do you feel?’ Anchors: 0 (not at all) and 10 (extremely). †, ‡, § means for the same outcome that have different symbols are significantly different (*p* < 0.05).

### 3.2 Differences in Hunger, Fullness and Desire to Eat According to Breakfast Portion Size

For this analysis, responses from 14 out of 16 participants were considered, as two participants did not fully complete the ordinal scales. Not all participants reported scores for 30AL and, therefore, this was not included in the analysis. For all three variables of hunger, fullness, and desire to eat, there was a significant main effect for breakfast (*p =* 0.003), time (*p <* 0.001), and breakfast by time (*p <* 0.001). The overtime analysis of the variables (Table 3) revealed that all three breakfasts had higher scores for hunger and desire to eat but lower for fullness for BB and BL than all the other time points (*p <* 0.001). Additionally, only after the small breakfast was the score for AL lower for hunger and higher for fullness compared to AB and 30AB (*p <* 0.05) but was not different for desire to eat (*p >* 0.05). For all three variables, there were no differences between BB and BL and AB and 30AB scores across the breakfast conditions (*p >* 0.05).

**TABLE 3:** Hunger, fullness, and desire to eat area under the curve (AUC) and ordinal scale ratings recorded at different time points for the small, standard and large breakfast in healthy individuals (*n* = 14).

Breakfast	BB	AB	30AB	BL	AL	AUC
Hunger						
Small	7.2 ± 1.4*	3.2 ± 2.2^‡^	3.1 ± 1.9^‡^	7.6 ± 1.6^*,‡^	0.6 ± 0.6^†^	8570 ± 2320^‡^
Standard	7.7 ± 1.2*	0.4 ± 0.5	1.1 ± 1.3	6.7 ± 2.2*	1.6 ± 1.5	6100 ± 1920
Large	7.6 ± 2.0*	0.5 ± 0.8	0.6 ± 0.8	5.6 ± 2.2*	0.7 ± 1.1	4843 ± 1891
Fullness						
Small	2.1 ± 2.2*	5.7 ± 2.5^‡^	5.5 ± 2.3^‡^	1.5 ± 1.3^*,§^	8.4 ± 1.2^†,¶^	7370 ± 2730^‡^
Standard	2.2 ± 2.3*	8.0 ± 1.4	7.4 ± 1.8	2.1 ± 1.6*	6.7 ± 2.1	9360 ± 2154
Large	1.0 ± 1.0*	9.1 ± 0.9	8.6 ± 1.2	3.5 ± 2.2*	7.8 ± 1.6	11 646 ± 2213¶
Desire to eat						
Small	6.9 ± 2.5*	2.5 ± 1.6^‡^	3.3 ± 1.9^‡^	7.7 ± 1.8^*,‡^	1.3 ± 1.6	8820 ± 2405^‡^
Standard	7.9 ± 1.3*	1.1 ± 1.6	1.4 ± 1.2^§^	6.7 ± 2.2*	1.9 ± 1.8	6500 ± 1975
Large	7.4 ± 2.1*	0.7 ± 1.2	0.6 ± 0.8	5.8 ± 2.6*	1.0 ± 1.2	5025 ± 2232

*Note:* Data are presented as group mean ± SD. BB (before breakfast), AB (after breakfast), 30AB (30 min after breakfast), BL (before lunch) and AL (after lunch). Hunger question: ‘How hungry do you feel now?’, anchors: 0 (not at all) and 10 (extremely); fullness question: ‘How full do you feel now?’, anchors: 0 (not at all) and 10 (extremely); desire to eat: ‘How strong is your desire to eat now?’, anchors: 0 (not at all) and 10 (extremely). For all the highlighted differences, *p* < 0.05 * different from AB, 30AB, and AL; ^†^ different from AB and 30AB; ^‡^ different from the other breakfasts; ^§^ different from the large breakfast; ¶ different from the standard breakfast.

Tests of difference (Table 3) between the three breakfasts at each time point showed that the small breakfast scores were higher for hunger and desire to eat between after breakfast and before lunch (AB, 30AB and BL) than the other breakfasts (*p <* 0.05). Moreover, the desire to eat also showed the standard breakfast had a higher score at 30AB compared to the large breakfast (*p <* 0.05). For fullness, the small breakfast scores at AB and 30AB were lower than the other breakfasts (*p =* 0.004). In addition, the BL score for fullness was lower for the small breakfast than the large breakfast (*p <* 0.05) but not the standard breakfast (*p >* 0.05). Additionally, for fullness, the AL score for the small breakfast was higher than the standard breakfast (*p <* 0.05) but not the large breakfast (*p >* 0.05), while AL scores were not different for hunger and desire to eat among the three breakfasts (*p >* 0.05). Across all the variables, no differences were detected among breakfasts for the BB scores (*p >* 0.05).

AUC analysis (Table 3) revealed that the desire to eat AUC was significantly higher for the small breakfast compared to the desire to eat AUC of the standard and large breakfasts (*p =* 0.004, *p* < 0.001, respectively), with no differences detected between the large and standard breakfasts (*p >* 0.05). The small breakfast fullness AUC was significantly lower compared to the fullness AUC of the standard and large breakfasts (*p* = 0.046, *p* < 0.001, respectively). Additionally, fullness AUC was higher for the large versus the standard breakfast (*p* = 0.028). The AUC for ratings of hunger was higher for the small breakfast compared to the standard and large breakfasts (*p =* 0.002, *p* < 0.001), while hunger AUC was not different between large and standard breakfasts (*p >* 0.05).

### 3.3 Energy Intake and Macronutrient Composition

One participant was excluded from this analysis for not providing the food diaries, resulting in data analysis from 15 participants. One female participant classed as normal weight did not finish the standard breakfast with 107 kcal not consumed, and 7 (5 females; 4 overweight, 2 normal weight, and 1 underweight) did not finish the large breakfast with an average of 41.5 ± 57.2 kcal not consumed. All the participants fully consumed the small breakfast.

One‐way repeated measures ANOVA revealed that the total daily energy intake without breakfast was significantly higher for the day of the small breakfast compared to the large breakfast (*p <* 0.05), but not for the standard breakfast (*p >* 0.05). Similarly, the energy consumed from snacks was higher on the day of the small breakfast than on the large breakfast (*p* < 0.05), but not for the standard breakfast (*p >* 0.05). No differences were detected for energy consumed at lunch, dinner and total daily energy intake with breakfast (*p >* 0.05). Data are shown in Table 4.

**TABLE 4:** Energy intake for lunch, dinner, snacks, total daily energy intake without breakfast and total daily energy intake with breakfast for small, standard and large breakfast in healthy individuals (*n* = 15).

	Small	Standard	Large
Lunch (kcal)	592.6 ± 157.7	486.8 ± 181.4	490.7 ± 121.3
Dinner (kcal)	604.3 ± 282.4	583.1 ± 236.4	531.7 ± 211.2
Snacks (kcal)	232.7 ± 157.1*	193.5 ± 144.9	120.6 ± 142.0
Total energy intake without breakfast (kcal)	1429.7 ± 395.3*	1263.4 ± 303.1	1143.0 ± 261.5
Total energy intake with breakfast (kcal)	1628.1 ± 399.9	1655.3 ± 299.1	1702.5 ± 278.4

*Note:* Data are presented as group mean ± SD.

* Different from the large breakfast (*p* < 0.05).

The macronutrient composition of lunch, dinner, snacks and total daily energy intake without breakfast, after the small, standard and large breakfast, is represented in Table 5. Analysis of snacks' macronutrient composition revealed a higher consumption of carbohydrates during the day after the small breakfast compared to the large breakfast (*p <* 0.01), with a similar trend seen for fat during the day after the small breakfast compared to the large breakfast (*p <* 0.05). No differences were found for protein (*p >* 0.05). Moreover, carbohydrate content in the total daily energy intake without breakfast was overall lower for the large breakfast day compared to small and standard (*p <* 0.05), while protein and fat content did not change (*p >* 0.05). There were also no differences in macronutrient intake for lunch and dinner after the three breakfast portions (*p >* 0.05).

**TABLE 5:** Macronutrient composition of lunch, dinner, snacks and total daily intake without breakfast after the small, standard and large breakfast in healthy individuals (*n* = 15).

	Small	Standard	Large
Lunch			
CHO (g)	79.1 ± 37.0	64.1 ± 38.7	60.0 ± 31.4
Protein (g)	27.9 ± 19.2	20.7 ± 9.4	20.4 ± 11.9
Fat (g)	16.9 ± 9.3	13.9 ± 10.0	16.9 ± 6.3
Dinner			
CHO (g)	59.6 ± 37.0	65.8 ± 34.0	52.7 ± 29.4
Protein (g)	31.3 ± 19.9	26.5 ± 14.7	28.2 ± 18.8
Fat (g)	22.1 ± 12.7	20.5 ± 12.4	18.9 ± 11.0
Snacks			
CHO (g)	30.0 ± 21.6**	23.3 ± 21.6	16.1 ± 17.9
Protein (g)	6.8 ± 6.6	3.5 ± 3.7	3.0 ± 4.1
Fat (g)	8.1 ± 6.9**	6.6 ± 4.9	3.2 ± 5.0
Total intake without			
CHO (g)	168.7 ± 57.5	153.3 ± 46.7	128.8 ± 40.6*
Breakfast			
Protein (g)	66.1 ± 38.9	50.8 ± 19.0	51.6 ± 28.1
Fat (g)	47.1 ± 17.2	41.0 ± 19.0	39.1 ± 11.3

*Note:* Data are presented as group mean ± SD. For all the highlighted differences, *p* < 0.05 *different from the small and standard breakfast **different from the large breakfast.

Abbreviation: CHO, carbohydrates.

## 4 Discussion

In this study, we explored how estimation of portion size and ‘expected satiety’ matched portion size and satiety evaluation upon consumption of different portions of the same breakfast and how the different portion sizes affected daily energy intake. We found that the small portion size of breakfast was considered the smallest and least filling of all three, while the large portion size was the largest and the most filling, with this consistent across evaluation of a visual image and after consumption of the breakfasts. When consuming the small breakfast, people reported being hungrier and with more desire to eat between breakfast and lunch and less full at 30 min after breakfast compared to the other breakfasts, but less full before lunch only compared to the large breakfast. Accordingly, fullness AUC was lower, and the desire to eat AUC and hunger AUC were higher for the small breakfast compared to the other breakfasts. Moreover, higher fullness scores were reported after lunch than after breakfast on the day of the small breakfast consumption. Along with a difference in satiety perception, we observed an increase in energy intake after lunch for the small breakfast compared to the large breakfast condition, interestingly due to more snacking with foods rich in carbohydrates and fat. Overall, the total daily energy intake was not significantly different among the three breakfast conditions, indicating that energy compensation occurred later in the day after the reduced breakfast size.

Our findings suggest that individuals can judge breakfast portion sizes similarly when presented visually or consumed. Haynes et al. (2019) achieved similar results after assessing visual perceptions of food portion sizes and their intended consumption. When portion sizes were ranked as small, participants indicated they intended to ‘compensate’ for the lack of energy intake, while when the portion sizes were considered as large, they reported they would consume only part of it (Haynes et al. 2019). Yet, Haynes et al. (2019) only evaluated the individuals' intention of consuming the food, while in the current study, we assessed portion size perception upon physical food consumption. Correspondingly, associations between visual portion size selection and subsequent consumption have been reported in a further study, which found that the visual estimated satiety matched both virtual and physical ideal portion size selection (Wilkinson et al. 2012). These findings are similar to our findings, where participants' scores for fullness were not different between visual and upon consumption, although in the Wilkinson et al. (2012) study participants selected and consumed only their ideal portion size, whereas in our study different portion sizes were given to participants for portion size estimation and consumption (Wilkinson et al. 2012).

Therefore, our work expands on previous research by demonstrating that estimated visual satiety and portion size are linked to meal consumption even when different portion sizes are imposed and are not self‐selected (Brunstrom 2014). Moreover, to our knowledge, our study is the first one to modulate breakfast portion sizes and to show that this manipulation does not affect individuals' satiety and portion size ‘matching’ ability.

The current study showed that after the consumption of the small breakfast, participants reported being hungrier, with more desire to eat and less full between breakfast and lunch compared to on the day of the large breakfast. We also found an increase in energy intake over the rest of the day after the small breakfast (286 kcal vs. large breakfast), which suggests that a 50% reduction in breakfast portion size might potentially lead to compensatory eating—in our case via snacking on foods rich in carbohydrates and fat. The compensatory effect (total energy intake without breakfast) observed in our study was attenuated for the standard (−167 kcal vs. small one) and large breakfasts (−120 kcal vs. standard one). These preliminary findings indicate that individuals tend to compensate to varying degrees during the day based on the size of the breakfast, resulting in no significant difference in the total daily energy intake across the three breakfast conditions. However, we cannot confirm that this compensatory response was due to being hungrier after lunch since we were unable to consistently collect ordinal scales at 30 min after lunch.

Currently, the effects of reducing portion size on daily energy intake are not clear. Some studies have shown that a reduction in meal portion size led to a diminished overall total daily calorie intake (Lewis et al. 2015; Haynes, Hardman, Halford, Jebb, Mead, et al. 2020), while others reported a compensatory effect (French et al. 2014; Haynes et al. 2019). The compensatory effect, seen in previous research and the current study, might be linked to the perception of portion sizes, specifically when perceived as smaller than ‘normal’. For instance, in the Haynes, Hardman, Halford, Jebb, and Robinson (2020) cross‐over study participants were served either a ‘large‐normal’, a ‘small‐normal’ or a ‘smaller than normal’ lunch portion, and they were allowed to help themselves with more of the same food if needed. An additional intake was higher for the ‘smaller than normal’ portion compared to the other portions (Haynes, Hardman, Halford, Jebb, and Robinson, 2020). However, this study only assessed the calorie intake of a meal and did not monitor daily calorie intake. When the same portion sizes were adopted for both lunch and dinner over 5 consecutive days, there was no influence of portion size perception on daily energy intake, despite an additional intake reported after lunch (Haynes et al. 2020). This suggests that the immediate availability of additional helpings of the manipulated portion size might be enough to compensate for the perceived smaller portion, with no additional intake needed throughout the day, contrary to the current and previous studies where additional immediate intake was not provided (French et al. 2014).

Another point to consider is that external influences, found in the real world, are able to impact eating behaviours (Gough et al. 2021). The current study and the study by French et al. (2014) were conducted in free‐living conditions and not in a laboratory setting, and this might have influenced the overall daily food intake (French et al. 2014). Additionally, this could potentially explain the difference in our findings compared to the study by Lewis et al. (2015), where breakfast portion sizes were also manipulated, yet further laboratory meals were provided (Lewis et al. 2015). Consequently, our results indicate that in a free‐living environment, without additional servings, individuals are prone to consume additional calories throughout the day following ingestion of a 50% reduction in breakfast portion size. It can be hypothesised that, in our study, a perceived reduction in breakfast portion size might have triggered a transient increase in food cravings for the remainder of the day (Meule 2020). Further research is needed to assess whether reduced portion sizes, both perceived and not perceived as normal, might influence energy intake in free‐living conditions and investigate the timeframe for possible ‘normalisation’ of perceived small portion sizes in the long term (Robinson and Kersbergen 2018).

In terms of appetite perception, we found that on the day of the small breakfast, an additional 100 kcal was consumed at lunch, and greater fullness was perceived after lunch. This suggests that a bigger lunch was ingested on that occasion, although it was not significantly different from the other lunches. On the other hand, the higher daily energy intake seen after the small breakfast, which resulted from more snacking between lunch and the end of the day, was significantly different compared to the other breakfast conditions. The most reported snacks were crisps, cake/biscuits, bread and crackers. Analysis of the macronutrient composition of snacks revealed that carbohydrates and fat were the main sources of energy. Among different snacking habits, it has been reported that morning snacking is linked to more consumption of nutrient‐dense items such as fruit and vegetables, whereas later snacking is linked to more energy‐dense items such as French fries and fast‐food meals (Barrington and Beresford 2019). The snacking reporting in this study was recorded from lunch onwards; therefore, there might have been the tendency to select more obesogenic types of snacks.

Consuming energy‐dense foods has been previously reported as compensatory eating for skipping or delaying breakfast and for smaller breakfast portion sizes (Santos‐Merx et al. 2017; Gwin and Leidy 2018; Wang et al. 2020). However, only one study monitored snack consumption by providing snacks to the participants, while in the current study, participants were free to choose their own snacks (Gwin and Leidy 2018). Therefore, our results add to the existing body of evidence by demonstrating that individuals prefer energy‐dense food after a small breakfast even under free‐living conditions. Increased snacking, with palatable food rich in carbohydrates and fat, especially combined, might potentiate reward and contribute to weight gain (Perszyk et al. 2021). Hence, more research investigating the role of snacking, as part of a compensatory response following smaller breakfast portion sizes, is paramount to further understand its impact on total daily energy intake.

Despite interesting preliminary findings, results from the current trial should be considered with caution as this was an exploratory study with a small sample size. The power calculation for this study was performed to detect differences in satiety and not total daily energy intake; therefore, although the total daily energy intake was smaller for the small breakfast than the large breakfast, the study sample might not have been large enough to detect significant differences. Moreover, graphic exposure to food stimuli can alter food consumption (Neyens, Aerts, and Smits 2015). In this study, how photographs of the three breakfasts were taken, including angle, distance from the plate and light conditions, might have contributed to an inaccurate perception of the plate size and the amount of food shown in the image, and as a consequence, rating (Nikolić et al. 2018). Further limitations of this study were the non‐exclusion of people on appetite control medications, the non‐inclusion of individuals living with overweight/obesity, and equal energy composition of the breakfasts for each individual. Several factors, such as gender, age, body size/composition and physical activity level, affect energy expenditure, which subsequently defines the energy requirements for each person (Redman et al. 2014). Yet, despite the amount of breakfast not being tailored based on personal energy requirements, it was based on healthy eating guidelines where the breakfast portion for an average adult should consist of 20% of the daily Reference Intake of 2000 kcal (Public Health England 2017; Gaal et al. 2018; British Nutrition Foundation 2021). Indeed, based on participants' responses to satiety and daily energy intake post‐breakfast consumption, the standard breakfast in our study appeared to be the most appropriate portion size for them. Further research should consider tailoring breakfast portion sizes to personal energy requirements. The present study was conducted during the COVID‐19 pandemic. The pandemic had both negative and positive impacts on dietary habits and lifestyle factors (Coulthard et al. 2021). In general, the diet quality of the majority of individuals improved as many individuals were cooking at home, using fresh produce (Bennett et al. 2021). Nevertheless, the pandemic also resulted in increased meal frequency and snacking, particularly of comfort foods such as sweets, processed and fried foods (Bennett et al. 2021). Furthermore, individuals with higher BMI who had tendencies to overeat prior to the pandemic were found to consume more high‐energy foods (Coulthard et al. 2021). All this might have had an impact on the energy intake of our participants; however, we believe that if this occurred, it would have influenced the three breakfast conditions equally.

## 5 Conclusion

In this study, individuals seemed to be accustomed to predicting satiety and portion size from visual images. Moreover, consuming half of the standard breakfast portion was judged not filling enough, and this was accompanied by a higher energy intake via snacking later in the day. Therefore, this study suggests breakfast portion size (and energy) reduction by 50% may lead to unhealthy compensatory energy intake by snacking on energy‐dense foods.

Future research is needed to confirm our exploratory findings. In particular, further studies in free‐living conditions should explore whether long‐term exposure to small portion sizes might lead to normalisation of smaller portions and whether this might influence energy intake in the long term. Additionally, the role of snacking as a compensatory response to reduction in breakfast portion sizes should also be investigated. More knowledge on whether breakfast portion size reduction might be a valid strategy to reduce daily energy intake and aid weight management is paramount, as this would better inform public health policies and practice.

## Author Contributions

**Kinga Kwiecien:** recruitment, data collection, formal analysis, and original draft preparation. **Lourdes Santos‐Merx:** conceptualization, methodology, formal analysis, writing, and editing. **Tarsem Sahota:** recruitment and editing. **Helen Coulthard:** writing and editing. **Mariasole Da Boit:** conceptualization, methodology, formal analysis, writing, and editing.

## Conflicts of Interest

The authors declare no conflicts of interest.

## Data Availability

The data that support the findings of this study are available from the corresponding author upon reasonable request.
